# Body composition is prognostic and predictive of ipilimumab activity in metastatic melanoma

**DOI:** 10.1002/jcsm.12538

**Published:** 2020-02-13

**Authors:** Michael P. Chu, Yuetong Li, Sunita Ghosh, Shelley Sass, Michael Smylie, John Walker, Michael B. Sawyer

**Affiliations:** ^1^ Cross Cancer Institute University of Alberta Edmonton Alberta Canada

**Keywords:** Ipilimumab, Melanoma, Myosteatosis, Body composition, Total body water

## Abstract

**Background:**

Body composition is minimally investigated in an immunotherapy era. Specific body composition signals such as myosteatosis may reflect aspects of patients' immunology and thereby their ability to respond to immunotherapies. Ipilimumab is a key checkpoint inhibitor in metastatic melanoma. As an antibody, it may also be more accurately dosed using body composition parameters rather than weight alone. This retrospective study aimed to investigate body composition‐based dosing and outcomes.

**Methods:**

Pretreatment computed tomography images from metastatic melanoma, ipilimumab‐treated patients from 2009 to 2014 were used to measure myosteatosis [skeletal muscle radiographic density or SMD, in Hounsfield units (HU)] and surface area (cm^2^) as previously described. Cut point analysis determined whether a level of ipilimumab dose and myosteatosis demonstrated differences in progression‐free (PFS) and overall survival (OS). Secondary endpoints included objective response rates and toxicities.

**Results:**

Of 121 identified, 97 patients were evaluable. Baseline demographics included 56 years median age, 60% male participants, and 23.7% with BRAF mutations. SMD analysis identified cut‐offs of SMD < 42 in those with BMI < 25 kg/m^2^ and <20 HU in those with BMI ≥ 25 kg/m^2^, respectively. Low SMD patients had poorer median PFS [2.4 vs. 2.7 months, hazard ratio (HR) 1.76, *P* = 0.008] and OS (5.4 vs. 17.5 months, HR 2.47, *P* = 0.001), which remained significant in multivariate modelling. High SMD patients had more immune‐related adverse events, better objective response rates (17.9 vs. 3.3%, *P* = 0.051), and lower baseline neutrophil‐to‐lymphocyte ratio (21 vs. 39%, *P* = 0.049). Separately, patients receiving <2.03 mg/cm^2^ had improved median PFS (3.0 vs. 2.6 months, HR 1.88, *P* = 0.02) and OS (14.9 vs. 5.7 months, HR 1.98, *P* = 0.01).

**Conclusions:**

Low SMD and receiving >2.03 mg/cm^2^ are prognostic of poorer melanoma outcomes post ipilimumab. SMD may identify patients with flawed immunology and predict who may better respond to such therapy. Ipilimumab dosing by skeletal muscle index stands in contrast to weight‐based dosing and may demonstrate a more accurate method of antibody dosing.

## Introduction

Melanoma is a highly immunogenic tumour based on its neoantigen load.^1^ This knowledge coupled with metastatic melanoma's (MM) previously poor prognosis served as the basis for studying immunotherapy. Ipilimumab, an anticytotoxic T‐cell lymphocyte antigen 4 (CTLA4) antibody, is thought to work by impeding inhibitory signals and allowing continued costimulatory interaction between B7 and CD28. In MM, ipilimumab improved both progression‐free (PFS) and overall survival (OS).^2^ Subsequently, other immune therapies have been shown to improve MM outcomes also including anti‐PD1 antibodies, such as nivolumab and pembrolizumab, either as monotherapy or in combination with ipilimumab.^3^, ^4^ With a significant number of effector cells, each with their own stimulatory and inhibitory signals, multiple therapeutic targets are now being studied.^5^

While these drugs have improved survival, finding predictive biomarkers for these therapies remains elusive. One example is PD1/PDL1 expression wherein multiple studies have reported its utility, but differing assays and cut‐offs makes these proteins' expression difficult to use clinically.^6^ Alternatively, body composition may be useful in this circumstance. While not focused upon specific targets, body composition studies a patient's composition by measuring volume and quality of muscle and adipose tissue compartments.^7^ Skeletal muscle is characterized on computed tomography (CT) with a range of density measured in Hounsfield units (HU). Muscle quality tends to be quite linear in that the lower radiographic density, the higher its fat content.^8^ When measured and averaged across a landmark, anatomic level, skeletal muscle density (SMD) has been shown to differentiate patients with more severe forms of diabetes and muscular dystrophy.^9^, ^10^ In fact, patients with low SMD not only have been shown to have poorer outcomes with these diseases, but low SMD has also been associated with higher circulating levels of inflammatory markers such interleukin‐6 and tumour necrosis factor.^11^

Low SMD is prognostic in renal cell cancer.^12^ We have demonstrated that low SMD is also prognostic in indolent and aggressive forms of lymphoma despite the use of rituximab, an anti‐CD20 monoclonal antibody.^13^, ^14^ In either study, response to rituximab‐containing, combination chemotherapy favoured those with high SMD. It is possible that low SMD in MM patients may predict for poorer responses to ipilimumab and be prognostic of these patients' outcomes. We investigated this hypothesis by retrospectively reviewing ipilimumab‐treated MM patients at our centre to determine if low SMD could prognosticate and/or predict survival and response.

Further, as proteins, monoclonal antibodies are highly charged molecules. By their nature, antibodies are exceedingly hydrophilic. Extrapolating that the majority of a patient's body mass is composed of water wherein the bulk of that water is housed in muscle^15^; monoclonal antibodies may be more appropriately dosed by total body water (as estimated by total muscle mass) rather than by either body surface area or body weight as antibodies are currently dosed. Rituximab may be an example of where dosing antibodies based on body surface area factors into why males have poorer outcomes.^16^ In this retrospective review, we also investigated whether ipilimumab dosing by muscle mass was predictive of response and prognostic of outcomes.

## Methods

### Patients

Following institutional ethics review approval, MM patients treated with ipilimumab single agent at a centralized, single institution of Northern Alberta (catchment population > 1.8 million) from 2009 through 2014 were reviewed. Patient demographics including age, stage, BRAF status at diagnosis, sex, height, weight, and performance status were collected. Dose, number of cycles received, and best response to ipilimumab were then documented. PFS and OS were collected as primary endpoints. Objective response rates (ORR), neutrophil‐to‐lymphocyte ratio, and treatment‐specific toxicities were secondary endpoints. Toxicities included for review included hospitalization, grades 3 or 4 diarrhoea, hepatitis (all grades as denoted by elevations in alanine aminotransferase and/or aspartate aminotransferase), dermatitis (all grades), and endocrine abnormalities (all grades).

### Body composition analysis

Height and weight at the first cycle of treatment was documented. Body composition was assessed using each patient's pretreatment, staging CT scans. To be included in review, CT scans must have been within 30 days prior to beginning therapy. To ensure image quality, CT scanner calibration was performed daily at start‐up using air in the CT scanner gantry then dynamically during scanning for individual patients using air as a negative control. Therefore, though CT tube current may fluctuate between patients, variability is minimal yielding the following CT parameters for each patient: contrast enhanced or unenhanced, 5 mm slice thickness, 120 kVp, and 290 mA. Two adjacent images at the L3 vertebral body level were used to measure total muscle surface area (cm^2^) and averaged. This vertebral landmark is chosen based on its linear correlation to total body lean body mass.^7^, ^17^ Muscles were quantified within a range of −29 to 150 HU using Slice‐O‐Matic software (version 5.0, TomoVision, Magog, Quebec, Canada).

Skeletal muscle radiographic density was quantified as mean muscle radiation attenuation (HU) of the muscle cross‐sectional area across the L3 vertebral body level as assessed between −29 and +150 HU.^8^

### Statistical analysis

The hypothesis is that a threshold value exists (cut point) of SMD and ipilimumab dose (based on muscle surface area) within these continuous variables that significantly increases progression and/or mortality risk. Such statistically defined cut points have been described in large populations of patients with solid tumours but not in patients treated with immune therapies.^18^ For that reason, this exploratory analysis in established threshold values within our population by a cut point analysis using minimal *P*‐value approach separately for SMD and ipilimumab dose.^19^ The continuous variable was divided based on each patient's defined SMD or ipilimumab dose and the cut point that provided maximum χ^2^ or provided minimum *P*‐value was chosen to be the point for dichotomizing the continuous variable.

Kaplan–Meier methods were used to compare PFS and OS between (i) groups of high vs. low SMD and (ii) high vs. low ipilimumab dose per cm^2^ of muscle. Survival was then compared by log‐rank test and multivariate Cox proportional hazards modelling using sex, age, lactate dehydrogenase (LDH), and BRAF status as covariates independently for SMD and ipilimumab dosing first then in combination. In both SMD and ipilimumab dosing groups, ORR and toxicities were compared by χ^2^ analysis employing a two‐sided *P*‐value. A similar approach was taken for comparison of neutrophil‐to‐lymphocyte (N:L) ratio and absolute lymphocyte count following two cycles of ipilimumab but specific for the SMD comparison. Statistical analysis was done using Statistical Analysis System (SAS, version 9.3 from SAS Institute Incorporated, Cary, North Carolina).

## Results

### Patients

Between 2009 and 2014, 121 MM patients received ipilimumab. Only 97 patients (80.2%) were included for this review because the remainder did not have a CT scan that fell within the prespecified time period prior to starting treatment. Median age was 56 years with a range from 25 to 91 years. Fifty‐eight patients were male (59.8%), 23 harboured a BRAF mutation (23.7%), and 30 had elevated circulating LDH levels at the time of treatment (30.9%). Eleven patients had locally advanced, unresectable disease (11.3%), while the remainder exhibited distant metastatic disease. Patients received a median two lines of therapy prior to ipilimumab (maximum was four) with 19 patients treated with ipilimumab frontline (19.6%). All patients were immunotherapy naïve. Twelve patients participated in a dose‐finding study wherein their doses remain blinded. Of the others, ipilimumab was given at 3 mg/kg in all except two who participated on a separate dose‐finding clinical trial (one received 0.3 mg/kg and the other, 10 mg/kg). Fifty‐one patients received four cycles of treatment (52.5%). Twenty patients (20.6%) were hospitalized while receiving ipilimumab. Seventy‐six patients (78.4%) reported gastrointestinal toxicities, of which 14 experienced grade 3 or 4 diarrhoea (14.4%). Patient characteristics are summarized in Table 1.

**Table 1:** Baseline patient characteristics, response and toxicities based on ipilimumab dose and SMD DCR, disease control rate; ECOG PS, Eastern Cooperative Oncology Group Performance Status; Ipi, ipilimumab; LDH, lactate dehydrogenase based on local laboratory definition; MSA, muscle surface area at L3 vertebral body level; ORR, objective response rates; SMD, skeletal muscle density; SMI, skeletal muscle index

Patient characteristic	All (*n* = 97)	Low SMD (33)	High SMD (*n* = 64)	*P*‐value (low vs. high SMD)	Ipi < 2.03 mg/cm^2^ (*n* = 62)	Ipi ≥ 2.03 mg/cm^2^ (*n* = 23)	*P*‐value (low vs. high dose)
Median age, years (range)	56 (25–91)	60 (29–87)	55 (25–91)	0.22	56 (25–91)	58 (30–87)	0.86
Sex, *n*				0.13			0.03
Male	58 (60.0%)	16 (48.5%)	42 (65.6%)		41 (66.1%)	9 (39.1%)	
Female	39 (40.0%)	17 (51.5%)	22 (34.4%)		21 (33.9%)	14 (60.9%)	
Stage, *n*				0.74			0.27
III	11 (11.3%)	3 (9.1%)	8 (12.5%)		10 (16.1%)	1 (4.3%)	
IV	86 (88.7%)	30 (90.9%)	56 (87.5%)		52 (83.9%)	22 (95.7%)	
Prior lines of therapy, *n*				0.07			0.81
1	19 (19.6%)	2 (6.1%)	17 (26.6%)		8 (12.9%)	3 (13.0%)	
2	59 (60.8%)	24 (72.7%)	35 (54.5%)		39 (62.9%)	17 (74.0%)	
3	15 (15.4%)	5 (15.2%)	10 (15.6%)		12 (19.4%)	3 (13.0%)	
4	4 (4.1%)	2 (6.1%)	2 (3.1%)		3 (4.8%)	0	
BRAF mutation, *n*				0.62			0.78
Negative	74 (76.3%)	24 (72.7%)	50 (78.1%)		17 (27.4%)	5 (21.7%)	
Positive	23 (23.7%)	9 (27.3%)	14 (21.9%)		45 (72.6%)	18 (78.3%)	
LDH, *n*				0.36			0.59
Normal	67 (69.1%)	25 (75.8%)	42 (65.6%)		45 (72.6%)	15 (65.2%)	
Elevated	30 (30.9%)	8 (24.2%)	22 (34.4%)		17 (27.4%)	8 (34.8%)	
ECOG PS, *n*				0.51			0.13
0	19 (19.6%)	9 (27.3%)	10 (15.6%)		11 (17.7%)	8 (34.8%)	
1	54 (55.6%)	18 (54.5%)	36 (56.3%)		45 (72.6%)	9 (39.1%)	
2	18 (18.6%)	5 (15.2%)	13 (20.3%)		14 (22.6%)	4 (17.4%)	
3	6 (6.2%)	1 (3.0%)	5 (7.8%)		4 (6.5%)	2 (8.7%)	
MSA, median cm^2^	135.0	134.7	137.7	0.71	141.7	109.7	0.23
SMI, median cm^2^/m^2^	47.4	44.4	48.7	0.67	48.9	40.0	0.19
ORR, *n*	12 (12.4%)	1 (3.0%)	11 (17.2%)	0.051	11 (17.5%)	0 (0 %)	0.03
DCR, *n*	31 (40.0%)	6 (18.2%)	25 (39.1%)	0.07	26 (41.9%)	15 (65.2%)	0.08
Hospitalizations, *n*	20 (20.7%)	4 (12%)	16 (25%)	0.19	12 (19.3%)	5 (21.7%)	0.76
Gastrointestinal toxicity, *n*	61 (62.9%)						
Grade 3 or 4	14 (14.4%)	3 (9.1%)	11 (17.2%)	0.21	7 (11.3%)	5 (21.7%)	0.28
Transaminitis, *n*	21 (21.6%)	2 (6%)	19 (30%)	0.008	13 (21.0%)	5 (21.7%)	0.98
Dermatitis, *n*	62 (63.9%)	12 (36%)	48 (75%)	0.003	34 (54.8%)	13 (56.5%)	0.89
Endocrinopathy, *n*	11 (11.3%)	3 (9%)	8 (13%)	0.74	9 (14.5%)	2 (8.7%)	0.72

### Skeletal muscle density

**Figure 1:**
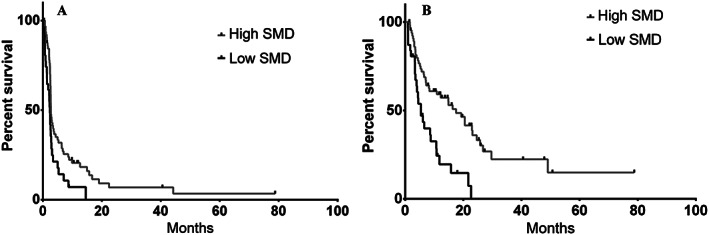
Kaplan–Meier survival curves based on baseline myosteatosis as measured by low skeletal muscle density (SMD) compared with high muscle composition (high SMD); (A) progression‐free survival and (B) overall survival.

Median SMD was 29.7 HU with a range of 3.7 to 49.4 HU. Cut point analysis revealed a substantial difference in both PFS and OS with an SMD value at 42 and 20 HU for nonoverweight (BMI < 25 kg/m^2^) and overweight (BMI ≥ 25 kg/m^2^), respectively. Low SMD was associated with poorer PFS compared with high SMD [median 2.4 vs. 2.7 months, 1 year 6.7 vs. 19.4%, HR 1.77, 95% confidence interval (CI) 1.12−3.31, *P* = 0.008, see *Figure* 1A]. OS was similarly significantly lower in low vs. high SMD patients (median 5.4 vs. 17.5 months, 2 year 0 vs. 33.9%, HR 2.47, 95% CI 1.84−6.02, *P* = 0.001, *Figure* 1B). It is worth noting that all deaths in this review were attributed to progression of disease.

When taking age, sex, line of therapy, LDH, and BRAF status into consideration in Cox proportional hazards modelling, low SMD retained its poor prognostic significance for OS (HR 2.12, 95% CI 1.17−3.85, *P* = 0.02, respectively; see *Table* 2).

**Table 2:** Univariate and multivariate analyses of variables assessing for impact on progression‐free (PFS) and overall survival (OS) based on baseline demographics and SMD

		Univariate analysis			Multivariate analysis		
	Variable	Hazard ratio	95% CI	*P*‐value	Hazard ratio	95% CI	*P*‐value
PFS	Low SMD	1.77	1.20–3.31	0.008	1.55	0.90–2.67	0.12
	Male sex				1.01	0.98–1.03	0.57
	Line of treatment (≥2)				1.21	0.68–2.16	0.52
	LDH (>ULN)				1.36	0.79–2.34	0.27
	Age (>60 years)				1.00	0.97–1.02	0.58
	BRAF mutation				1.60	0.89–2.86	0.11
OS	Low SMD	2.47	1.84–6.02	0.001	2.12	1.17–3.85	0.02
	Male sex				1.21	0.71–2.47	0.70
	LDH (>ULN)				1.80	1.00–3.36	0.05
	Line of treatment (≥2)				1.54	0.79–2.66	0.24
	Age (>60 years)				1.01	0.99–1.03	0.47
	BRAF mutation				1.77	0.95–3.31	0.07

CI, confidence interval; LDH, lactate dehydrogenase; SMD, skeletal muscle density; ULN, upper limit of normal.

ORR trended to favour high SMD patients (17.9 vs. 3.3%, *P* = 0.051). When also considering stable disease, high SMD patients also trended toward better disease control rates (38.8 vs. 20.0%, *P* = 0.07). While rate of hospitalization, grade 3 or 4 colitis, and endocrinopathies did not statistically differ between high vs. low SMD patients, rates of any hepatitis/transaminitis (30 vs. 6%, *P* = 0.008) and dermatitis (75 vs. 36%, *p* = 0.003) were significantly higher in high SMD patients (see *Table* 1).

Presence of low SMD is hypothesized to represent an overall, higher inflammatory state. Based on recent evidence suggesting that overall inflammation in MM patients may also be represented by an elevated N:L ratio,^20^ patient's baseline, preipilimumab N:L ratio was compared between high and low SMD patients. Baseline N:L was elevated in a higher proportion of low SMD patients (39 vs. 20%, *P* = 0.049). Elevated absolute lymphocyte count following two cycles of ipilimumab has also been suggested as being both predictive and prognostic of OS in MM patients.^21^ Absolute lymphocyte counts were significantly higher in high vs. low SMD patients in this review also (1.3 vs. 0.9 × 10^9^ cells/L, *P* = 0.036), though it should be noted that five patients were excluded in this calculation owing to having received only one cycle of ipilimumab.

### Ipilimumab dose by muscle surface area

**Figure 2:**
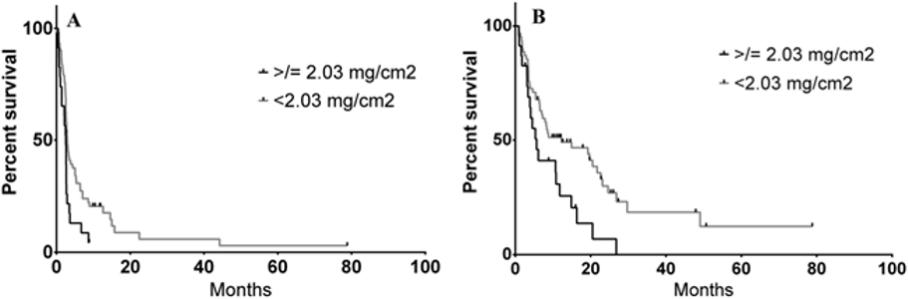
Kaplan–Meier survival curve based on ipilimumab dosing as a function of actual dose received per muscle surface area (cm^2^) measured at the third lumbar vertebrae on computed tomography imaging; (A) progression‐free survival and (B) overall survival.

Of the 85 patients evaluable for this analysis, median muscle surface area at the L3 vertebral body level was 135.9 cm^2^ among all patients reviewed (range 62.2 to 228.5 cm^2^). To determine ipilimumab dose concentration per patient, actual dose received was divided by muscle surface area because of the latter's linear correlation with total body mass.^7^ In doing so, median ipilimumab dose concentration was 1.76 mg/cm^2^ with a range of 0.16 to 4.84 mg/cm^2^. By cut point analysis, a significant difference in PFS and OS was denoted between patients who received <2.03 mg/cm^2^ compared with ≥2.03 mg/cm^2^ (*n* = 23. 27.1% of this population). Demographics between patients who received above or below this threshold were very similar (see *Table* 1). Median PFS comparing these two dosing groups found a detriment to those who received ≥2.03 mg/cm^2^ (2.5 vs. 2.92 months, HR 1.88, 95% CI 1.23−3.99, *P* = 0.01, *Figure* 2A). Median OS similarly demonstrated a detriment for patients receiving ipilimumab above this cut point (5.7 vs. 12.2 months, HR 1.98, 95% CI 1.25−4.39, *P* = 0.05, *Figure* 2B).

In multivariate Cox proportional hazards modelling, being dosed ≥2.03 mg/cm^2^ retained its prognostic significance for both PFS (HR 2.28, 95% CI 1.23−4.09, *P* = 0.004) and OS (HR 2.53, 95% CI 1.41−4.93, *P* = 0.002, see *Table* 3) when also considering advanced age, male sex, LDH, positive BRAF status, and sarcopenia. Sarcopenia was included because of its inherent potential to confound dose by muscle surface area and was defined by usual parameters.^18^ Not surprisingly, sarcopenia had prognostic implications on PFS and OS (see *Table* 3). ORR favoured patients receiving doses under the ipilimumab muscle‐dose cut point (17.5 vs. 0%, Fisher's *t*‐test, *P* = 0.03). No difference in rate of hospitalization (21.7 vs. 19.4%) or hepatitis (21.7 vs. 21.0%) was observed between high and low dosing groups (see *Table* 1). A statistically insignificant higher incidence of grade 3 or 4 gastrointestinal toxicities occurred in the higher dosing group (21.7 vs. 11.3%, *P* = 0.23). Though presence of elevated N:L ratio was no different between the two groups, higher absolute lymphocyte count following two cycles of ipilimumab trended in favour of lower dosing.

**Table 3:** Univariate and multivariate analyses of variables assessing for impact on progression‐free (PFS) and overall survival (OS) based on baseline demographics and ipilimumab dosing

		Univariate analysis			Multivariate analysis		
	Variable	Hazard ratio	95% CI	*P*‐value	Hazard ratio	95% CI	*P*‐value
PFS	Dose (≥2.03 mg/cm^2^)	1.88	1.23–3.99	0.01	2.28	1.23–4.09	0.004
	Sarcopenia				1.85	1.06–3.22	0.03
	Male sex				1.12	0.71–2.00	0.38
	Line of treatment (≥2)				1.58	0.95–2.79	0.09
	LDH (>ULN)				1.21	0.73–1.99	0.46
	Age (>60 years)				1.03	0.94–1.07	0.43
	BRAF mutation				2.54	1.33–4.93	0.02
OS	Dose (≥2.03 mg/cm^2^)	1.98	1.25–4.39	0.05	2.53	1.41–4.93	0.002
	Sarcopenia				2.46	1.35–4.51	0.004
	Male sex				1.02	0.58–1.70	0.98
	Line of treatment (≥2)				1.39	0.79–3.31	0.15
	LDH (>ULN)				1.67	0.95–2.90	0.08
	Age (>60 years)				1.00	0.98–1.03	0.85
	BRAF mutation				1.61	0.90–2.92	0.10

CI, confidence interval; LDH, lactate dehydrogenase; ULN, upper limit of normal.

In a separate multivariate modelling, ipilimumab dosing and SMD were combined with advanced age, male sex, positive BRAF status, and LDH in 85 patients with known ipilimumab dose. Low SMD continued to significantly impact PFS and OS (HR 1.78, 95% CI 1.02−3.09, *P* = 0.04 and HR 2.46, 95% CI 1.35−4.51, *P* = 0.004, respectively). Receiving ipilimumab ≥2.03 mg/cm^2^ also retained its prognostic impact on PFS and OS (HR 2.75, 95% CI 1.54−4.88, *P* = 0.001 and HR 2.86, 95% CI 1.53−5.38, *P* = 0.0004, respectively; see *Table* 4).

**Table 4:** Multivariate analysis combining SMD and ipilimumab dose based on muscle surface area

		Multivariate analysis		
	Variable	Hazard ratio	95% CI	*P*‐value
PFS	Dose (≥2.03 mg/cm^2^)	2.75	1.54‐4.88	0.001
	Low SMD	1.85	1.06‐3.22	0.03
	Male sex	1.39	0.86‐2.27	0.18
	Line of treatment (≥2)	1.17	0.67‐2.06	0.58
	LDH (>ULN)	1.39	0.82‐2.35	0.22
	Age (>60 years)	1.01	0.99‐1.04	0.60
	BRAF mutation	1.81	0.99‐3.30	0.05
OS	Dose (≥2.03 mg/cm^2^)	2.86	1.53‐5.38	0.001
	Low SMD	2.46	1.35‐4.51	0.004
	Male sex	1.41	0.79‐2.53	0.25
	Line of treatment (≥2)	1.67	0.81‐3.41	0.16
	LDH (>ULN)	1.66	0.93‐2.97	0.09
	Age (>60 years)	1.01	0.99‐1.03	0.38
	BRAF mutation	1.96	1.05‐3.69	0.04

CI, confidence interval; LDH, lactate dehydrogenase; OS, overall survival; PFS, progression‐free survival; SMD, skeletal muscle density; ULN, upper limit of normal.

## Discussion

Fatty infiltration of muscle, or myosteatosis, as demonstrated by low SMD appears to occur in more severe forms of disease where it is also associated with increased circulating cytokine levels.^11^ While myosteatosis denotes poorer prognosis in other malignancies such as lymphomas,^14^ this study is among the first to demonstrate its prognostic and predictive applicability for patients treated with immunotherapy. Moreover, we observed an association between baseline myosteatosis and underlying inflammation with higher N:L ratios. Taken together, we speculate that myosteatosis may be predictive of ipilimumab response because of its ability to discern patients with severely altered immune systems. This hypothesis would also help to explain the significant ipilimumab‐specific toxicities experienced by patients without myosteatosis, presuming these individuals retain a relatively intact immune system. Strikingly, the durable responses seen with ipilimumab appears to be restricted to those individuals with high SMD (*Figure* 2). Given the potential severe toxicities associated with ipilimumab, utilization of SMD as a predictive biomarker may be of tremendous utility, particularly as the information is readily discernible from standard‐of‐care, baseline diagnostic imaging. The presence of both myosteatosis and lower absolute lymphocyte count after two cycles of treatment may be more coincidental given their independent association with response as opposed to a true association.

Despite connections between general inflammation and myosteatosis, what remains unclear is how this affects the direct tumour microenvironment. A study by Malietzis *et al*. suggests that an association exists between dendritic cell function and body composition.^22^ In this prospective study of colorectal cancer patients, presence of low SMD was significantly associated with poorer expression of CD83 and CCR7, a costimulatory/maturation and migration signal, respectively, on circulating dendritic cells. Lacking these two important signals, these cells would be far less efficient at antigen presentation and activation of T‐cells. The authors also found an association with higher dendritic cell CD36 expression in patients with higher SMD suggesting that the opposite is true with improved antigen collection and presentation in these patients. Others have also demonstrated how lipids can accumulate in cancer patients' dendritic cells leading to their dysfunction.^23^ This work draws an interesting and important connection between immune function and myosteatosis, a phenomenon that occurs preferentially in patients with cancer as opposed to those who are simply obese.

The concept of using body composition as a means of more accurately determining treatment dose is similar to work done to improve carboplatin dosing based on renal function.^24^ It is worth noting that analysing muscle surface area and radiation attenuation (SMD) in fact analyse two separate facts regarding a given patient. An analogy would be to describe the physical dimensions of an object separate from its density. In this way, both muscle surface area and SMD have been found to be independently prognostic in other cancers.^18^ This rationale is also supported in our multivariate analysis finding that myosteatosis and ipilimumab dosing have independent impact on melanoma patient survival (*Table* 4). Further, antibodies are charged proteins, which should make them exceedingly hydrophilic. As such, antibody dosing should be more reliant on total body water rather than body weight or body surface area. Aside from circulating blood volume, the predominant focus of body water is found within muscle.^25^ Circulating volume is relatively similar between patients, but muscle volumes vary greatly. Measuring muscle surface area on CT imaging at L3 vertebral body level carries exceedingly high agreement with actual total body measurements.^7^ Rather than introducing further estimations by using calculations to derive total body water, and therefore higher degrees of error, we calculated ipilimumab dose directly on measured muscle surface area at this vertebral body level. In doing so, ipilimumab doses in fact are spread over a wide degree of concentrations. Interestingly, patients who received a lower ipilimumab dose based on muscle surface area (and by extension, total body water) had better PFS and OS. Consequently, we posit that it may be more rational and effective to dose antibodies based on total body water via measuring muscle surface area rather than traditional methods of body weight or body surface area. MM has provided an interesting population to examine because of the use of single agent antibodies compared with either lymphoma or breast cancer where rituximab and trastuzumab are typically used in combination with cytotoxic chemotherapy, respectively. Subsequent investigations are focused at looking at whether this dosing scheme also yields similar findings in these populations with combination treatment. It is worth noting though that therapeutic antibody activity is naturally influenced by many factors beyond hydrophilicity such as tumour heterogeneity, target antigen density, blood flow to tumour(s), pharmacokinetics, and pharmacodynamics.^26^ Further, the body composition definitions established in this study had to veer from previously defined criteria for low SMD and MSA because of the poor body composition of MM patients.

A limitation by virtue of a retrospective design is the inability to test for and compare circulating cytokine levels between patients with or without myosteatosis. Ongoing prospective studies are aimed at specifically looking at circulating cytokine levels and whether or not patients with myosteatosis may have differences within tumour microenvironments and muscle. Further, an artefact may be present given the lack of long term survivors in the low SMD and higher dose/MSA groups likely as a fact of the relatively small sample size. While this study focused ipilimumab dosing based on muscle surface area, work into creating more accurate estimations of total body water are underway to take into consideration the slight variations in circulating volume and the small proportion of adipose tissue that is still water. Recent trial data have found that combined ipilimumab and anti‐PD1 treatment to be more effective than single agent therapy.^3^ As such, whether these ipilimumab findings can be applied in combination therapy or to PD‐1 antibodies in general remains an area for further investigation.

## Conflict of interest

All authors declare no conflicts of interest.

## Funding

No funding was provided for this study.
